# Queen Recognition Signals in Two Primitively Eusocial Halictid Bees: Evolutionary Conservation and Caste-Specific Perception

**DOI:** 10.3390/insects10120416

**Published:** 2019-11-21

**Authors:** Iris Steitz, Katharina Brandt, Felix Biefel, Ädem Minat, Manfred Ayasse

**Affiliations:** Institute of Evolutionary Ecology and Conservation Genomics, University of Ulm, 89069 Ulm, Germany; katjbrandt@gmail.com (K.B.); felix.biefel@gmx.de (F.B.); aedem_minat@hotmail.de (Ä.M.); manfred.ayasse@uni-ulm.de (M.A.)

**Keywords:** chemical communication, sweat bees, queen signals, Halictidae, queen control vs. queen signal hypothesis

## Abstract

Queen signals are known to regulate reproductive harmony within eusocial colonies by influencing worker behavior and ovarian physiology. However, decades of research have resulted in the identification of just a few queen signals, and studies of their mode of action are rare. Our aim was to identify queen recognition signals in the halictid bee *Lasioglossum pauxillum* and to analyze caste differences in the olfactory perception of queen signals in *L. pauxillum* and the closely related species *L. malachurum*. We performed chemical analyses and bioassays to test for caste differences in chemical profiles and worker behavior influenced by queen-specific compounds in *L. pauxillum*. Our results indicated that caste differences in the chemical profiles were mainly attributable to higher amounts of macrocyclic lactones in queens. Bioassays demonstrated a higher frequency of subordinate behavior in workers elicited by queen-specific amounts of macrocyclic lactones. Thus, macrocyclic lactones function as queen recognition signals in *L. pauxillum*, as in *L. malachurum*. Using electrophysiological analyses, we have demonstrated that queens of both tested species lack antennal reactions to certain macrocyclic lactones. Therefore, we assume that this is a mechanism to prevent reproductive self-inhibition in queens. Our results should stimulate debate on the conservation and mode of action of queen signals.

## 1. Introduction

Eusocial insect colonies of termites, ants, wasps, and bees are mainly characterized by their complex behavior, including the reproductive division of labor between reproductive queens and nonreproductive or sterile workers [1]. The effectiveness of such colonial living is mainly attributable to highly evolved communication systems. In particular, chemical signals are known to be involved not only in mediating interactions of individuals and in colony organization, but also in the regulation of reproduction [2,3,4]. Indeed, chemical signals derived from egg-laying females are thought to affect the fertility and reproductive success of workers in many eusocial insect taxa and are therefore key components in maintaining their colonial success [3,4,5,6,7].

However, decades of research with the aim of revealing the role of queen chemical compounds as signals that influence worker reproduction have only resulted in the chemical identification of ‘real’ queen signals in a few social insect species [6]. Simple long-chain cuticular hydrocarbons (CHCs) have been shown to exert an effect on the behavior of workers or an inhibitory effect on their reproductive physiology by inducing sterility or inhibiting the ovarian development of workers. Such effects have been investigated in several ant species (*Lasius niger*, *L. flavus*, *L. lasioides*, *Cataglyphis iberica*, *Odontomachus brunneus*, and *Aphaenogaster cockerelli*), the common wasp *Vespula vulgaris* and the Saxon wasp *Dolichovespula saxonica*, the stingless bee *Friesella schrottkyi*, and the bumblebee *Bombus terrestris* [6,8,9,10,11,12,13,14,15]. Additionally, a comparative study across more than 60 different social insect species has indicated that fertile females exhibit, in general, a higher amount of long-chain CHCs than nonfertile females [15]. Based on their own experiments and data from the literature, the authors suggest that CHCs act as a conserved class of queen signals across several independently evolved eusocial lineages of insects. However, this hypothesis is mainly based on comparative statistical analyses of chemical data, and specific bioassays to establish their function as queen signals are often missing. Despite this, several additional studies have shown that compounds other than CHCs have a function as queen signals in, for example, the honeybee *Apis mellifera* and the termite *Reticulitermes speratus* [16,17,18]. Moreover, chemical analyses and bioassays in various trap-jaw ant species of the genus *Odontomachus* have revealed a more complex picture, with CHCs and other compounds probably functioning synergistically as queen signals, although these signals are also more divergent among the different species of the same genus [19]. Indeed, various species of *Odontomachus* have been shown to exhibit different chemical compounds as fertility signals characterizing the queens, which indicates that queen signals are even not conserved within this genus [19]. However, most of these studies dealing with queen signals have focused on species with a highly derived eusocial behavior and obvious caste-specific differences in the composition of chemical compounds. The study of queen signals in these species therefore seems to be less meaningful in terms of investigating the evolutionary transitions of eusociality. A more suitable approach would be to examine species on the transition from solitary behavior to eusociality, such as halictid bees or sweat bees (Hymenoptera: Halictidae). Indeed, a recent study on a primitively eusocial halictid bee species, *Lasioglossum malachurum*, has clearly demonstrated that macrocyclic lactones, not CHCs, act as a queen signal influencing workers behavior and physiology [20], thereby rejecting the hypothesis of a conserved signal across all eusocial insects. Additionally, a comparative study involving several halictid species with various social levels has indicated that macrocyclic lactones are the most prominent compounds in fertile breeding queens compared with workers in this taxon [21]. However, more studies including bioassays ascertaining the behavioral and physiological impact of these compounds on species additional to *L. malachurum* are needed to definitively establish whether macrocyclic lactones function as queen signals across several halictid bee species.

In halictid bees, macrocyclic lactones are of considerable interest over many years of research. They are very abundant and highly diverse among a wide range of halictid bees [22]. They are produced in large amounts in the Dufour’s gland and the primary function is to line the inner surface of brood cells and nest entrances with a hydrophobic and germicide lining against fungi and microorganisms [23,24,25,26]. However, they can be also found on the cuticle surface and evolved diverse functions in various communication systems, including sex pheromones [27]; but see [28,29,30], size- and dominance-signaling among gynes competing for suitable nesting sites [31], and queen recognition and queen signaling [20].

In addition to the question of the chemical identity of queen signals in eusocial insects, the mechanism by which these chemical signals influence worker reproduction is still under discussion. Debate continues as to whether queen-produced chemicals directly influence and manipulate worker behavior and physiology (‘queen control hypothesis’) or are an informative honest signal for the presence and fertility of the queen, indirectly influencing workers to forego their own reproduction because of their higher indirect fitness value (‘queen signal hypothesis’) [4,32]. As a consequence of queen control against the interests of workers, an evolutionary arms race between queens and workers should evolve as workers try to overcome the queen’s manipulation and produce their own male offspring; this would result in a high complexity of queen signals among social insect species [4,32]. Despite this, queens must have a mechanism to prevent self-inhibition by their own pheromones, potentially through a lack of suitable receptors [4]. Indeed, several studies on eusocial insect species have revealed caste-specific qualitative and quantitative differences in antennal sensilla [33,34,35]. However, nowadays, the more prominent hypothesis is the queen signal hypothesis, as a reliable honest signal of queen’s quality, will result in a more stable evolutionary framework [6,36,37,38]. Most studies addressing this issue can be interpreted both ways, and an empirical way of discriminating between the two hypotheses is difficult to find [38].

In this study, we have focused on two primitively eusocial halictid bee species: *L. pauxillum* and a close relative, namely the well-studied species *L. malachurum*. Both species are small to tiny sweat bees distributed all over the Western Palearctic and are very abundantly found in Germany. The two species nest at barely vegetated habitats, the nests of *L. pauxillum* being mainly characterized by a soil chimney above the nest entrance. The life cycle of both species is typical for primitively eusocial halictid bees. A single overwintered female founds a nest in spring and provisions the first brood consisting of workers on her own. After the workers emerge, the queen remains within the nest and focuses on egg laying, whereas the workers take over all other tasks including brood care, nest building, and foraging. The last brood of the year consists of males and gynes. The gynes mate and overwinter to become foundresses the next year, whereas all other individuals die [39].

In this context, we tested whether we could find similar patterns regarding caste-specific chemical signals and queen recognition in *L. pauxillum* as previously known for *L. malachurum* [20]. As queen recognition is thought to be linked to queen signaling [40], the results of our study should contribute to ongoing research on the chemical identity of queen signals. Specifically, we have explored whether queens and workers differ in their cuticle odor bouquets, which substances mainly cause these differences, and the way that these compounds are involved in caste-specific recognition mechanisms. An understanding of these mechanisms underlying intercaste communication systems is a major precondition for further explaining worker sterility and overall the evolution of eusocial behavior in the Halictidae. Moreover, we have also analyzed the queen and worker perception of various odor compounds found on the queen’s cuticle in the two species, *L. pauxillum* and *L. malachurum*. These results have been used to check whether queen and worker antennae exhibit different odorant receptors in order to determine whether queens can perceive their own signals and to shed more light on the mode of action of queen signals. Our hypotheses were that queens and workers of *L. pauxillum* would differ in their cuticle chemical profiles and that queens would exhibit higher amounts of certain compounds (potentially also macrocyclic lactones), similar to the results previously found in *L. malachurum* [20]. Additionally, we assumed that these signals would function as a queen-recognition signal influencing worker behavior. Such comparative studies dealing with chemical signals among closely related species should elucidate the possible conservation of queen signals within halictid bees.

## 2. Materials and Methods

### 2.1. Bee Collection

All tested *L. pauxillum* and *L. malachurum* females were collected at a huge nesting aggregation in Ulm-Mähringen (Germany) by using an insect net or a vacuum suction device as described in [41]. Nest foundresses, breeding queens, and workers for chemical analyses were collected in spring and summer 2015, with all bees being individually placed in small plastic vials (Eppendorf tubes: 0.5 mL) and killed by freezing at −40 °C for further use. Nest foundresses and breeding queens were distinguished by the time of collection (nest foundresses in March/April, breeding queens in June/July) and the developmental stage of their ovaries (nest foundresses with undeveloped, breeding queens with developed ovaries). Queens and workers used for the electrophysiological analyses or circle tube bioassays were collected in summer 2016, 2017, and 2018. All bees were individually placed in plastic vials and chilled down immediately to approximately 4 °C in an ice box in the field. After being transferred to the lab, the bees were stored at 8 °C in single plastic vials (Eppendorf tubes: 0.5 mL) containing moist paper for a maximum of 36 h before electrophysiological analyses or bioassays were performed.

### 2.2. Chemical Analyses

Frozen females were individually rinsed for 15 s in 200 µL n-pentane (Uvasol, 99.5%, Merck, Germany) to extract cuticle surface compounds. All extracts were concentrated under a gentle stream of nitrogen to 25% of their initial volume. For quantitative analysis, 10 µL n-octadecane (stock solution: 100 µg/mL in n-hexane) was added to each extract as an internal standard. We pooled cuticle extracts of queens (N = 3 for each species) to further use in the electrophysiological analyses.

Chemical analyses were performed on an Agilent 7820 A Series gas chromatograph (Agilent Technologies, Waldbronn, Germany) equipped with a non-polar DB-5 MS capillary column (30 m × 0.25 mm inner diameter, J&W, New Berlin, WI, USA) and a flame ionization detector (GC-FID) with hydrogen as the carrier gas (constant flow, 2.0 mL/min). One microliter of each sample was injected splitless into the gas chromatograph (injector temperature: 310 °C), operating at 50 °C for 1 min, after which the split valve was opened, and the temperature was increased continuously by 10 °C/min to a final temperature of 310 °C. The structural elucidation of individual compounds was performed with a HP 6890 gas chromatograph (Hewlett Packard, Germany) connected to a mass selective detector (GCMS; Quadrupol 5972, Agilent, Santa Clara, CA, USA) using the method as described above for GC (carrier gas: helium). It was based on comparisons of mass spectra by using references from the NIST11 library and GC retention times with those of authentic reference samples by using AMDIS 2.71 (Automated Mass Spectral Deconvolution and Identification System). The absolute amounts of all substances were determined by using the GC-FID data analyzed with Agilent ChemStation Software (Agilent Technologies, Waldbronn, Germany) and the internal standard (i.e., n-octadecane) as a reference. In order to estimate relative proportions for further downstream analyses, absolute amounts of individual compounds were divided by the sum of the absolute amounts of all compounds and multiplied by 100. These results have previously been published in our comparative chemical study in halictid bees [21].

### 2.3. Measurement of Physiological State

After extraction of the cuticle surfaces, all bees were dissected under a stereomicroscope to check for ovarian stage and caste, which was classified into five categories according to [42].

### 2.4. Electrophysiological Analyses

Electrophysiological analyses were performed on an Agilent 7820 A Series gas chromatograph (Agilent Technologies, Waldbronn, Germany) with a flame-ionization detector (FID) and an EAD setup (Syntec, Hilversum, The Netherlands). For each GC-EAD run, one antenna of a *L. pauxillum* or *L. malachurum* queen or worker was cut off at its basis and at the tip of the excised antenna and mounted between two glass-capillaries filled with insect Ringer solution (0.42 g KCl, 5 g NaCl, 0.125 g CaCl in 1 L demineralized water). The recording electrode at the tip of the antenna was connected via an amplifier to a signal interface board (Syntech, Hilversum, The Netherlands) of a PC. The analyses were performed on an unpolar DB-5 capillary column (30 m × 0.25 mm inner diameter, J&W, New Berlin, WI, USA). Two microliters of the pooled queen cuticle extract were injected splitless into the gas chromatograph (injector temperature: 310 °C), operating at 50 °C for 1 min, after which the split valve was opened, and the temperature was increased continuously by 10 °C/min to a final temperature of 310 °C. The effluent was split (Gerstel outlet splitter, split ratio FID:EAD 1:1), and the outlet for the EAD was placed in a cleaned and humidified airflow that was directed over the antenna to prevent dehydration. The outlet was heated (310 °C) to avoid condensation of the effluent in the cooler airflow. EAD and FID signals were recorded simultaneously on a PC running a GC-EAD program (Syntech, Hilversum, The Netherlands). We considered a substance to be EAD-active when it proved to be active in a minimum of four replicates.

### 2.5. Circle Tube Bioassays

To investigate the role of different compounds on worker-queen behavior in *L. pauxillum*, we conducted several behavioral observations in circle tube arenas in the laboratory similar to those already performed with *L. malachurum* females [20]. For each bioassay, we used two bees from the same nest and marked one of them individually on the thorax using a small dot of water-based paint. Before starting the tests, the bees were allowed to warm up for 15 min. Afterwards, the two bees were placed into a silicon tube (length of 10 cm and an inner diameter of 4 mm, similar to the natural nest diameter) simultaneously from opposite ends before the tube was molded to a circle. The behavior of both interacting bees was observed for 10 min (starting after the first encounter) under red light and a constant room temperature of 26 °C. We focused on five different behavioral traits, as seen in Table 1.

We observed the behavior of workers when interacting with their queen or with a nestmate worker in five different trials: (1) worker vs. queen; (2) worker vs. worker; (3) worker vs. worker coated with queen cuticle extract; (4) worker vs. worker coated with a synthetic mixture of queen-specific amounts of macrocyclic lactones; and (5) worker vs. worker treated with pure solvent (pentane) as a control. In contrast to our study on *L. malachurum* [20], we did not test n-alkanes in *L. pauxillum* as the chemical analyses revealed no clear higher amounts of these compounds in breeding queens compared to workers.

For conducting bioassays with workers coated with queen cuticle extracts, queens were killed by freezing at −20 °C after using them for previous bioassays (trial 1). Cuticle surface compounds were extracted by rinsing the queens for 15 s in 200 μL of n-pentane (Uvasol, 99.5%, Merck, Germany). The synthetic mixture of macrocyclic lactones was blended by using the differences between queens and workers in total amounts of the substances on their cuticles as calculated with previously published results [21]. One bee equivalent corresponded to 0.459 µg 18-octacosanolide, 0.295 µg 20-eicosanolide, 0.132 µg 22-docosanolide, and 0.025 µg 24-tetracosanolide. All macrocyclic lactones were synthesized and provided by Prof. Wittko Francke (University of Hamburg, Hamburg, Germany).

To coat the bees, we inserted the extracts or the synthetic mixtures (corresponding to one bee equivalent) into single plastic vials (0.5 mL, Eppendorf tubes). We allowed the solvent to evaporate completely, and additionally waited for 30 min to prevent any harmful impact of the solvent toward the bees. Subsequently, each bee was individually placed for 30 min in one of the plastic vials containing the compounds, a cotton tip being used to prevent them from escaping. The bees were carefully forced to move and turn in the plastic vial, so that they smeared the compounds sticking to the plastic vials onto their own cuticle [43]. After 30 min, the coated bee was immediately used to perform a bioassay.

### 2.6. Statistics

Cuticular odor bouquets of eight replicates per female life stage and caste (i.e., nest foundresses, workers, and breeding queens) were tested for chemical dissimilarities. Relative amounts (%) of each compound were calculated with respect to the total concentration of the odor bouquet, and peaks with a concentration <0.1% were excluded from downstream analyses. To visualize chemical dissimilarities, we performed nonmetric multidimensional scaling (NMDS) based on Bray–Curtis square root transformed values as implemented in Primer [44]. Based on the same resemblance matrix, we performed a one-way ANOSIM (analysis of similarities, permutations: 10,000) following post-hoc SIMPER to check for relative contributions of certain compounds to caste-specific or life-stage-specific differences.

The frequency of each behavior observed in the circle tube tests was calculated as the number of times a certain behavior was performed divided by the total number of frontal encounters in each trial. Mann–Whitney U tests were used to analyze differences in the behavioral pattern between (i) queens and workers, (ii) workers and nestmate workers and to compare the worker behavior when interacting with either a queen or a worker. For further comparisons of the behavioral pattern of a worker when interacting with another worker before and after the coating of the opponent bee, we used Wilcoxon signed rank tests. All statistical analyses and illustrations were performed with SPPS v. 20.0 for Windows (SPSS Inc., Chicago, IL, USA) and Adobe Illustrator CC v.18.0.0 and R v. 3.1.1 [45].

## 3. Results

### 3.1. Caste-Specific and Female-Function-Specific Odor Profiles in L. pauxillum

**Figure 1:**
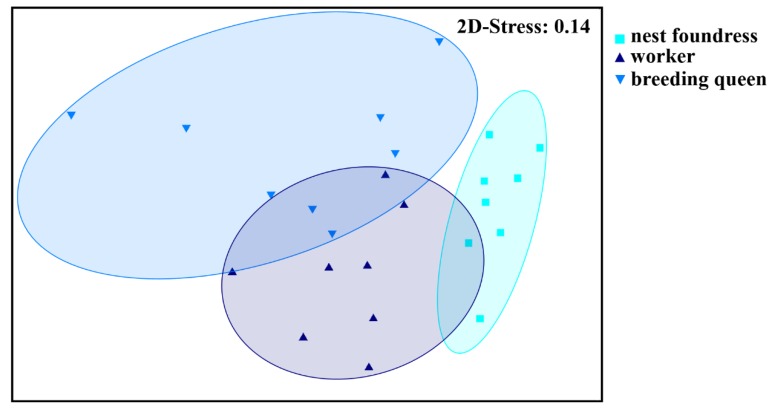
Differences in cuticular chemical profiles among nest foundresses, workers, and breeding queens of the halictid bee *L. pauxillum* (NMDS, Bray–Curtis similarity measures, 2D-Stress: 0.14). All tested female groups could be separated on the basis of the relative amounts of the chemical compounds found on their cuticle surfaces (ANOSIM, global R = 0.493, *p* < 0.001, foundresses vs. breeding queens: *p* < 0.001, foundresses vs. workers: *p* < 0.001, breeding queens vs. workers: *p* = 0.015).

In total, we found 79 different chemical compounds in the cuticle surface extracts of our tested *L. pauxillum* females (table of compounds published in [21]); these compounds mainly belonged to the typical sweat bee compound classes of n-alkanes, n-alkenes, saturated and unsaturated macrocyclic lactones, saturated and unsaturated isopentenyl esters, and ethyl esters [21,28,46,47]. All tested female groups, i.e., nest foundresses, breeding queens, and workers, possessed the same chemical compounds on their cuticle but exhibited significantly different odor profiles because of quantitative differences of these compounds (ANOSIM, global R = 0.493, *p* < 0.001, all pairwise comparisons: *p* < 0.05; Figure 1). The main compounds separating nest foundresses from breeding queens were the n-alkanes tricosane and nonacosane, the n-alkene (*Z*)-9-heptacosene, the macrocyclic lactones 18-octadecanolide and 20-eicoanolide, the isopentenyl ester 3-methyl-3-butenyl tetracosanoate, and seven unknown compounds (SIMPER analyses, each compound contributed more than 2.0% to the total Bray–Curtis dissimilarity; Figure S1). Despite this, breeding queens and workers were mainly separated by the relative amounts of the n-alkanes tricosane, pentacosane, heptacosane and nonacosane, the n-alkenes (*Z*)-9-pentacosene, (*Z*)-9-heptacosene, and (*Z*)-9-nonacosene, the macrocyclic lactones 18-octadecanolide, 20-eicosanolide, 22-docosanolide and 24-tetracos-(*Z*)-11-enolide, the isopentenyl ester 3-methyl-3-butenyl tetracosanoate, and six unknown compounds (SIMPER analyses, each compound contributed more than 2.0% to the total Bray–Curtis dissimilarity; Figure S2). Breeding queens were shown to have a higher relative amount of the macrocyclic lactones and the isopentenyl esters, whereas workers had higher relative amounts of n-alkanes and n-alkenes, particularly regarding those n-alkanes with higher chain lengths (>C_23_; [21]).

### 3.2. Queen–Worker Interactions and Queen Recognition in L. pauxillum

**Figure 2:**
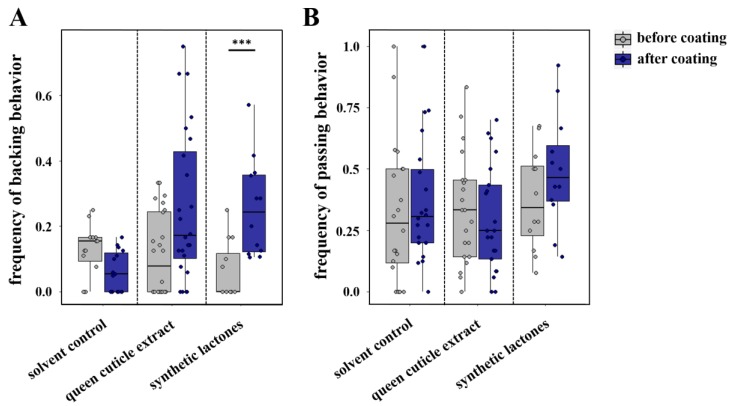
Comparison of *L. pauxillum* worker (**A**) backing behavior and (**B**) passing behavior when interacting with another worker before coating (gray) or after coating (blue) of the other worker. Bars represent the median as well as the 25th and 75th percentiles (***: significant difference, Wilcoxon signed rank test, *p* < 0.001).

Our circle tube bioassays performed with *L. pauxillum* females revealed a significantly higher frequency of passing behavior when workers interacted with other workers compared with the interactions with queens (Mann–Whitney U test, *p* < 0.001; Figure S3). In contrast, workers exhibited a significantly higher frequency of backing behavior when interacting with their queens compared with any interaction with another worker (Mann–Whitney U test, *p* = 0.011; Figure S3), similar to the results previously found in *L. malachurum* [20]. In our further bioassays, we checked whether a coating with queen cuticle extract, queen-specific amounts of macrocyclic lactones, or a solvent control could influence these two behaviors. The results indicated that the coating of a worker with the queen cuticle extract tended to elicit a higher frequency of backing behavior in the interacting worker compared with the frequency before the coating (Wilcoxon signed rank test, *p* = 0.054; Figure 2A), but coating with the synthetic mixture of macrocyclic lactones alone was sufficient to elicit a significantly higher frequency of backing behavior in the interacting worker (Wilcoxon signed rank test, *p* < 0.001; Figure 2A). This higher frequency was similar to the frequency of backing behavior shown by workers when interacting with their queens (Mann–Whitney U tests, *p* > 0.05). In contrast, coating with pentane as a solvent control had no significant effect on the frequency of backing behavior (Wilcoxon signed rank tests; *p* = 0.064; Figure 2A). With regard to passing behavior, we could not find any significant differences of the interacting worker before or after coating with any extract or with the solvent control (Wilcoxon signed rank tests; queen cuticle extract: *p* = 0.594; solvent control: *p* = 0.299; synthetic lactones: *p* = 0.206; Figure 2B).

### 3.3. Caste-Specific Perception of Odor Signals

**Figure 3:**
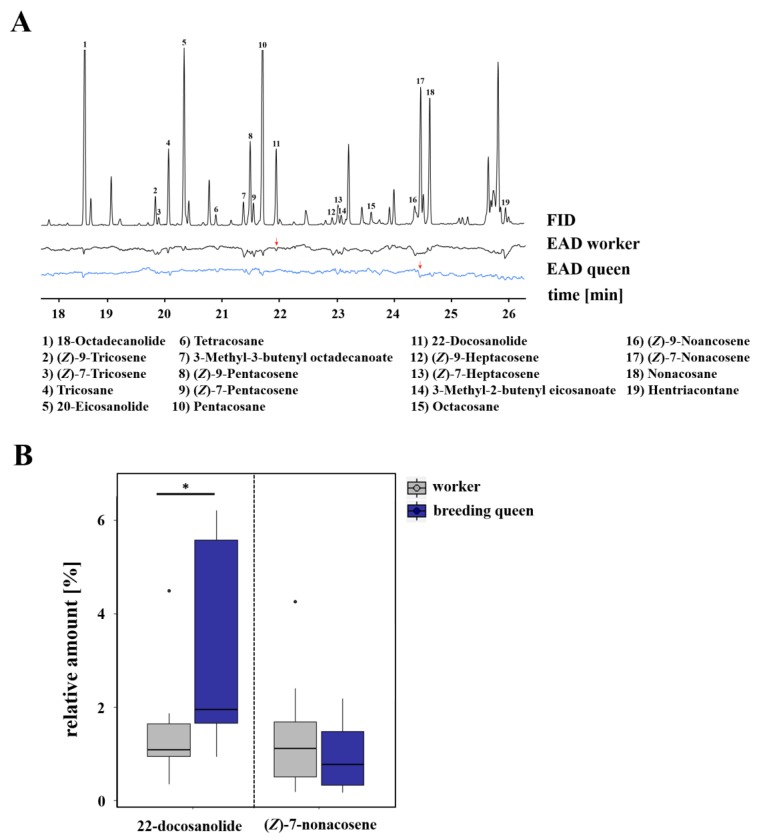
(**A**) Examples of coupled gas chromatographic and electroantennographic detection (GC-EAD) in cuticle surface extracts of *L. pauxillum* queens by using antennae of *L. pauxillum* workers (black) and queens (blue). Numbers indicate compounds that were electrophysiologically active (EAD-active) in at least four runs of workers or queens. Red arrows indicate those compounds that were perceived only by either workers or queens. For a better graphical presentation, antialiasing was performed on the baseline by using Adobe Illustrator CC v.18.0.0. (**B**) Comparison of relative amounts of those compounds which were only perceived by workers or queens. Bars represent the median as well as the 25th and 75th percentiles (*: significant difference, Mann–Whitney U test, *p* = 0.021).

With regard to the cuticular chemical profile of breeding queens in *L. pauxillum*, we identified a total of 19 compounds as being electrophysiologically active compounds that were perceived either by workers or queens. The perceived compounds were six n-alkanes (i.e., tricosane, tetracosane, pentacosane, octacosane, nonacosane, hentriacontane), eight n-alkenes (i.e., (*Z*)-9-tricosene, (*Z*)-7-tricosene, (*Z*)-9-pentacosene, (*Z*)-7-pentacosene, (*Z*)-9-heptacosene, (*Z*)-7-heptacosene, (*Z*)-9-nonacosene, (*Z*)-7-nonacosene), three saturated macrocyclic lactones (i.e., 18-octadecanolide, 20-eicosanolide, 22-docosanolide), and two saturated isopentenyl esters (i.e., 3-methyl-3-butenyl octadecenoate, 3-methyl-2-butenyl eicosanoate; Figure 3, Figure S4). However, the n-alkene (*Z*)-7-nonacosene was shown to be electrophysiologically active in the antennae of queens, but not in the antennae of workers. On the other hand, the macrocyclic lactone 22-docosanolide was only electrophysiologically active in workers’ antennae, but not in the antennae of queens, as seen in Figure 3A. Comparing the relative amounts of these compounds, breeding queens were shown to have a significantly higher relative amount of 22-docosanolide (Mann–Whitney U test, *p* = 0.021) than workers, whereas breeding queens and workers had the same relative amounts of (*Z*)-7-nonacosene (Mann–Whitney U test, *p* > 0.05; Figure 3B).

**Figure 4:**
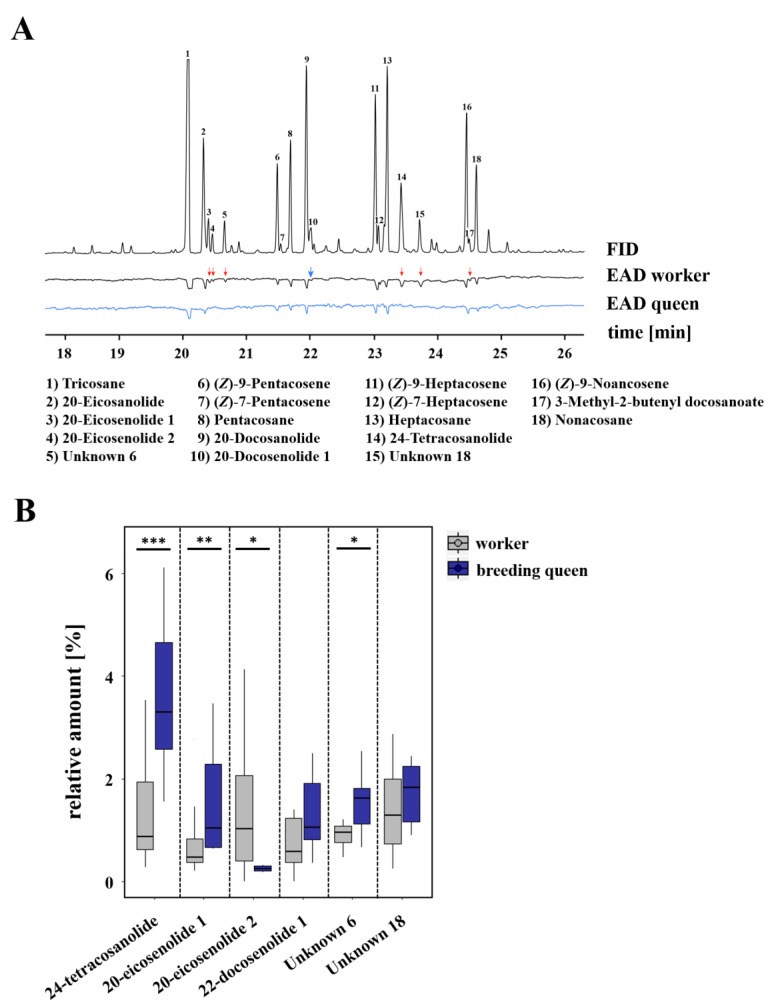
(**A**) Examples of coupled gas chromatographic and electroantennographic detection (GC-EAD) in cuticle surface extracts of *L. malachurum* queens by using antennae of *L. malachurum* workers (black) and queens (blue). Numbers indicate compounds that were electrophysiologically active (EAD-active) in at least four runs of workers or queens. Red arrows indicate those compounds that were perceived only by either workers or queens. For a better graphical presentation, antialiasing was performed on the baseline by using Adobe Illustrator CC v.18.0.0. (**B**) Comparison of relative amounts of those compounds which were only perceived by workers or queens. Bars represent the median as well as the 25th and 75th percentiles (asterisks indicate significant differences, Mann–Whitney U tests: 24-tetracosanolide *p* < 0.001, 20-eicosenolide 1 *p* = 0.007, 20-eicosenolide 2 *p* = 0.011, Unknown 6 *p* = 0.023).

Furthermore, 18 of the electrophysiologically active compounds that we found in the cuticular chemical profile of *L. malachurum* queens were either perceived by workers only or by both castes. These electrophysiological active compounds included four n-alkanes (i.e., tricosane, pentacosane, heptacosane, nonacosane), five n-alkenes (i.e., (*Z*)-9-pentacosene, (*Z*)-7-pentacosene, (*Z*)-9-heptacosene, (*Z*)-7-heptacosene, (*Z*)-9-nonacosene), three saturated macrocyclic lactones (i.e., 20-eicosanolide, 22-docosanolide, 24-tetracosanolide), three unsaturated macrocyclic lactones (i.e., two 20-eicosenolides and one 22-docosenolide with unknown double bond positions), one saturated isopentenyl ester (i.e., 3-methyl-2-butenyl docosanoate), and two unknown compounds, as seen in Figure 4 and Figure S5. The saturated macrocyclic lactone 24-tetracosanolide, all three unsaturated macrocyclic lactones, the isopentenyl ester 3-methyl-2-butenyl docosanoate, and both unknown compounds could only be perceived by workers antennae, but not by queens, as seen in Figure 4A. Comparing the relative amounts of these compounds, breeding queens were shown to have a significantly higher relative amount of 24-tetracosanolide (Mann–Whitney U test, *p* < 0.001), 20-eicosenolide 1 (Mann–Whitney U test, *p* = 0.007) and one unknown compound (Mann–Whitney U test, *p* = 0.023) than workers, whereas workers had a significantly higher relative amount of 20-eicosenolide 2 (Mann–Whitney U test, *p* = 0.011; Figure 4B).

## 4. Discussion

Our results clearly demonstrate caste-specific and female-life-stage-specific differences in the cuticle odor bouquets of *L. pauxillum* females mainly attributable to different relative amounts of certain n-alkanes, n-alkenes, saturated macrocyclic lactones, and an isopentenyl ester. Additionally, the typical behavior of workers when interacting with a queen is a high frequency of backing behavior, similar to the results found for worker behavior in *L. malachurum* [20]. This high frequency of backing behavior also occurs when a worker interacts with another worker coated with queen-specific amounts of macrocyclic lactones. Therefore, the behavior of a worker when interacting with a lactone-treated worker is similar to the typical worker–queen behavior, as has also previously been demonstrated for *L. malachurum* [20]. These results show that macrocyclic lactones play a crucial role in eliciting subordinate behavior of workers in *L. pauxillum* and therefore serve as queen recognition signals in two different primitively eusocial halictid bee species, namely *L. malachurum* and *L. pauxillum*. Moreover, we have shown that workers also exhibit a higher passing frequency when interacting with another worker than with a queen. Interestingly, this behavior does not change when the interacting worker is coated, either with the queen cuticle extract or with the queen-specific amounts of macrocyclic lactones. Additionally, we have demonstrated a differential perception of queen-produced signals among queens and workers in the two species, *L. pauxillum* and *L. malachurum*. This is commonly shown by the lack of queen antennal reactions to macrocyclic lactones.

In terms of the exploration of variable chemical communication systems in sweat bees, macrocyclic lactones have been of considerable interest over many years of research [21,27,28,31,47]. One reason for this interest is their frequency in a wide range of sweat bee species [22]. In addition to their preliminary function of forming a hydrophobic layer to protect the nest tubes and brood cells [23,24,25,26], they have also evolved diverse functions in various communication systems, including sex pheromones [27]; but see [28,29,30], size- and dominance-signaling among gynes competing for suitable nesting sites [31], and queen recognition and queen signaling [20]. In our study, we have clearly demonstrated that macrocyclic lactones are highly expressed in *L. pauxillum* breeding queens compared with workers, a result that is consistent among several other halictid bee species [21]. As a queen-specific quantity of macrocyclic lactones is also able to elicit subordinate behavior in workers, as is usually shown in worker–queen interactions, we assume that macrocyclic lactones are queen recognition signals in *L. pauxillum*, as in *L. malachurum* [20]. A definitive proof that they also function as queen signals influencing worker’s ovarian activation awaits further bioassays, as described in [20].

However, with regard to the chemical profiles of *L. pauxillum* queens and workers, we have also obtained an interesting pattern for n-alkanes. Breeding queens show a higher quantity of n-alkanes with shorter chain-lengths compared with workers (i.e., C17–C22), whereas workers exhibit a larger amount of those n-alkanes with longer chain-lengths compared with queens (i.e., C23–C31; [21]). The n-alkanes with shorter chain-lengths do not appear to contribute much to the differences in odor profiles among castes. Because CHCs are mainly used for desiccation resistance in insects [48], the higher amount of CHCs on the cuticles of workers can be explained on the basis of their outside activities in summertime when temperatures are high. Queens forage for just a few weeks in early spring and cease to leave their nests after provisioning the first brood of the year, indicating a lower demand for desiccation resistance and, consequently, a smaller amount of CHCs. Indeed, the CHC profiles of insects have previously been shown to depend on climatic conditions, whereas a higher temperature leads to higher amounts of CHCs [49]. This might also be the reason for the higher amount of especially long-chain CHCs in workers compared with queens, because CHCs with a higher chain length are known to provide better waterproofing for the insect and therefore better drought resistance [49,50]. This could also explain the demand of a higher amount of CHCs in nest foundresses compared to breeding queens, which was also previously shown for *L. malachurum* [46], as breeding queens, which cease to leave their nests, should have a lower demand for desiccation resistance than nest foundresses, even if they forage in early spring, where temperatures are not as high as in summer time.

Interestingly, all n-alkanes have been shown to be expressed to a greater extent in queens compared with workers in the closely related species *L. malachurum* with respect to both relative and absolute amounts [20]. In addition, n-alkenes have been shown to be expressed to a greater extent in workers compared with queens in both species. In other eusocial insect species, n-alkanes and especially n-alkenes have been shown to function as queen signals among almost all investigated species so far [6,8,9,10,11,12,13,14,15]. Indeed, CHCs are thought to be linked to the ovarian development in insects, and as queen signals may have evolved from byproducts of the ovarian activation, they might function as queen signals possibly across all social insects [6,15,51]. However, some sweat bee species seem to lack such a link between CHCs and ovarian development, as *L. pauxillum* workers with no developed ovaries have even higher amounts of CHCs compared with the breeding queens. Therefore, we still assume that queen signals have evolved from chemical compounds that were present in solitary ancestors and that may be directly linked to the fertility of females as suggested in [37]. However, we suggest that it is better not to focus only on CHCs, as other compounds might have evolved with a direct link to fertility [21]. As macrocyclic lactones are primarily used for nest building in sweat bees [23,24,25,26], they are indeed linked to fecundity and fertility and may therefore have easily evolved into queen signals in this taxon. Comparative studies involving the analysis of solitary and social breeding females of facultatively eusocial species are necessary, including, for example, manipulation experiments, in order to elucidate this problem.

Beside this, it was shown that behavioral interactions of the queens, e.g., aggressive behavior towards workers, is necessary to induce sterility in workers in some primitively eusocial insect species, whereas chemical signals are not sufficient to affect worker’s reproductive physiology [52,53,54,55,56,57]. Regarding our recent study on *L. malachurum*, we could clearly demonstrate that macrocyclic lactones as a chemical signal of the queen are solely sufficient to induce sterility in workers [20]. Furthermore, *L. pauxillum* queens never exhibited any aggressive behavior towards their workers in our circle tube arenas, which leads to the assumption that physical queen control over workers is also unlikely to occur in *L. pauxillum*. However, our recent comparative study on caste differences in cuticular chemical profiles among several halictid bee species with various social levels demonstrated, that castes of facultative eusocial species are chemically less distinct than castes in obligate eusocial species [21]. These smaller caste differences could be a hint for other signals to be involved in the regulation of worker’s reproduction, potentially also aggressive behavior as was shown in other primitively eusocial species such as *Polistes* wasps and bumble bees [52,53,54,55,56,57]. However, more bioassays are needed to prove, if and how other than chemical signals play a role in regulating worker’s reproduction in different halictid bee species.

Regarding female-life-stage-specific chemical differences, we found that nest foundresses and breeding queens differ in their chemical profiles. Indeed, chemical distances between breeding queens and workers on the one hand, and nest foundresses and breeding queens on the other hand, are similar among several halictid bee species [21], indicating that all female-life-stages exhibit a unique chemical odor profile, which was already shown before in *L. malachurum* [46]. Therefore, we suggest a necessity to always consider the life-stage of a queen when studying any caste differences in eusocial insects, as the production of chemicals with a communicative function in interactions with workers should be higher in breeding queens that have direct contact with workers than in nest foundresses, especially as the production of certain pheromones might be quite costly.

Our circle tube tests have revealed that a high frequency of backing behavior is a typical subordinate or queen-avoiding behavior of workers, a result that has previously been shown for other sweat bee species [20,58]. In *L. malachurum*, backing behavior always occurs immediately after a nudging event of the queen, but also frequently when the worker is in close proximity to the encountering individual without any previously aggressive interaction. This result indicates that the typical subordinate worker behavior is partially an aggression-avoidance behavior mediated by chemical signals that can be detected when another individual is close-by [20]. However, even without any specific nudging behavior of queens in *L. pauxillum*, we still assume that the backing behavior is a subordinate behavior elicited by perceiving the presence of a dominant female, namely the queen. Regarding the passing behavior, we found that workers pass other workers significantly more often than their queen, a result which was previously also shown for another primitively eusocial halictid bee [59]. However, such a decrease in passing behavior could not be repeated when the encountering worker was impregnated with the queen cuticle extract or queen-specific amounts of macrocyclic lactones. Therefore, we assume that the passing behavior of workers is, in contrast to the backing behavior, less influenced by the chemical compounds characterizing both castes. While the backing behavior is a very fast movement, which occurs immediately when a worker is in close proximity to a queen or a worker coated with macrocyclic lactones, the passing behavior usually occurs at the end of an encounter. Therefore, individual characteristics other than chemical cues, such as the size of a female, could influence passing behavior, especially the behavior of an encountering bee. As coating a worker with queen-specific amounts of chemical compounds did not change the behavior of this worker, we assume that they still behave like workers and not like queens. Therefore, we suggest that passing behavior is influenced synergistically by different characteristics of the encountering bee and not solely by chemical signals as the backing behavior. However, this hypothesis still needs to be proven by more suitable bioassays.

The results of our electrophysiological analyses have revealed differences in the perception of queens and workers in both the tested species, namely *L. pauxillum* and *L. malachurum*. In both species, queens were not able to perceive certain macrocyclic lactones. Moreover, in *L. pauxillum*, workers showed no antennal reactions to n-alkenes, leading to the assumption that castes differ in their physiological or morphological structure of their antennae. Similar results have previously been obtained for other hymenopteran species and are mainly explained by qualitative or quantitative differences in the antennal sensilla [33,34,35]. Moreover, studies on the weaver ant *Oecophylla smaragdina* have indicated that the variation of sensilla in the different castes are caused by differential development in terms of the number of primary mother cells, the precursors of chemosensory structures in the antennae [33]. Moreover, queens of *A. mellifera* are known to have a lower volume of olfactory glomeruli, and a smaller number of synapses within the olfactory center of the brain has been linked to a lower ability to perceive or learn the function of chemical signals [60]. Therefore, caste-specific differences in the structures of the antennae or the olfactory center of the brain are widespread among insects; unfortunately, discussions of the function of these differences are missing in most studies. In our study, we have shown that workers, but not queens, can perceive certain macrocyclic lactones, although the specific compound is dependent on the species. In addition, a mixture of macrocyclic lactones has been demonstrated to be a queen recognition signal in both species; however, we still do not know whether only a single compound or several of the tested compounds synergistically act to signal the queen’s presence. If the specific macrocyclic lactones, which could not be perceived by the queen of the tested species, are the main compounds functioning as a queen recognition signal and potentially also influencing worker ovarian development, the lack of antennal reactions to this compound by the queens provide new insights into the mechanisms by which queen signals actually act. With regard to the debate of the queen control hypothesis vs. the queen signal hypothesis, the former is only consistent when queens prevent any self-inhibition by their pheromone [4]. A lack of receptors in the antennae of the queens might prevent such self-inhibition. Thus, our findings are consistent with the queen control hypothesis. However, the queen signal hypothesis does not completely exclude the existence of mechanisms to prevent self-inhibition by the queen. Therefore, our results can be interpreted as evidence for both hypotheses. Hence, further neurophysiological analyses of the glomeruli and additional bioassays focusing on subsets or single macrocyclic lactones are needed to shed more light on this subject.

## 5. Conclusions

The results of our chemical analyses and bioassays have revealed that *L. pauxillum* females use macrocyclic lactones as a queen recognition signal, whereas a higher queen-specific amount of these compounds is sufficient to elicit subordinate or avoiding behavior in workers without any previous queen-like aggressive behavior, similar to the results previously found in *L. malachurum* [20]. Even if CHCs act as a conserved class of queen pheromones in various social insect lineages [6,10,15], sweat bees seem to have evolved macrocyclic lactones as queen signals [20]. As queen signals are thought to have evolved from fertility-linked cues present in solitary ancestors [15,37,61], sweat bees might have followed the same evolutionary strategies as ants, wasps, or other bees when evolving their queen signal. However, they do not use CHCs, but rather macrocyclic lactones, which are clearly linked to nest-building activities and therefore fertility and fecundity in sweat bees. Since this is the second study indicating that macrocyclic lactones function as queen recognition signals and potentially also as queen signals influencing workers reproduction, and because macrocyclic lactones seem to be expressed to a greater extent in queens compared with workers among several sweat bee species [21], we hypothesize the conservation of macrocyclic lactones as queen signals in the Halictidae. However, further studies, including bioassays testing the influence of macrocyclic lactones on ovarian activation in *L. pauxillum* and other sweat bee species, are necessary and should stimulate new debates on the evolution of the chemical identity and mechanisms of queen signals.

## Figures and Tables

**Table 1:** Behaviors recorded in circle tube bioassays.

Behavior	Description	Mode of Interaction
backing	fast movement backwards apart from the encountering bee	avoidance/submission
moving backwards	slow movement backwards apart from the encountering bee	avoidance
nudging	fast movement forwards, bee brings its face into contact with face of encountering bee	dominance
passing	both encountering bees pass and move on in opposite directions	cooperation
withdraw	180 degree turn away from encountering bee	avoidance

## Supplementary Materials

The following are available online at https://www.mdpi.com/2075-4450/10/12/416/s1, Figure S1: Comparison of relative amounts of (a) n-alkanes and macrocyclic lactones and (b) the n-alkene and isopentenyl ester which contributed more than 2.0% to the Bray–Curtis dissimilarities between nest foundresses and breeding queens, Figure S2: Comparison of relative amounts of (a) n-alkanes, (b) macrocyclic lactones and (c) n-alkenes and isopentenyl ester which contributed more than 2.0% to the Bray–Curtis dissimilarities between workers and breeding queens, Figure S3: Comparison of worker behavior interacting with another worker or a queen, Figure S4: Examples of coupled gas chromatographic and electroantennographic detection (GC-EAD) in cuticle surface extracts of *L. pauxillum* queens by using four different antennae of *L. pauxillum* workers and queens, Figure S5: Examples of coupled gas chromatographic and electroantennographic detection (GC-EAD) in cuticle surface extracts of *L. malachurum* queens by using four different antennae of *L. malachurum* workers and queens.
