# Supplementary material for: Queen Recognition Signals in Two Primitively Eusocial Halictid Bees: Evolutionary Conservation and Caste-Specific Perception
Source: Insects. 2019 Nov 21;10(12):416. doi: 10.3390/insects10120416 (PMC6955767; doi:10.3390/insects10120416)
Supplement: Supplementary file 1 [file insects-10-00416-s001.pdf]

Supplementary Material

Insects – Special Issue: Semiochemicals and Insect Behavior

Queen recognition signals in two primitively eusocial halictid bees: evolutionary conservation and caste-specific perception

Iris Steitz*[1], Katharina Brandt[1], Felix Biefel[1], Ädem Minat[1], Manfred Ayasse[1]

[1] *Institute of Evolutionary Ecology and Conservation Genomics, University of Ulm, 89069 Ulm, Germany*

**Correspondence: iris.steitz@gmx.net, (+49) 731-5022663*

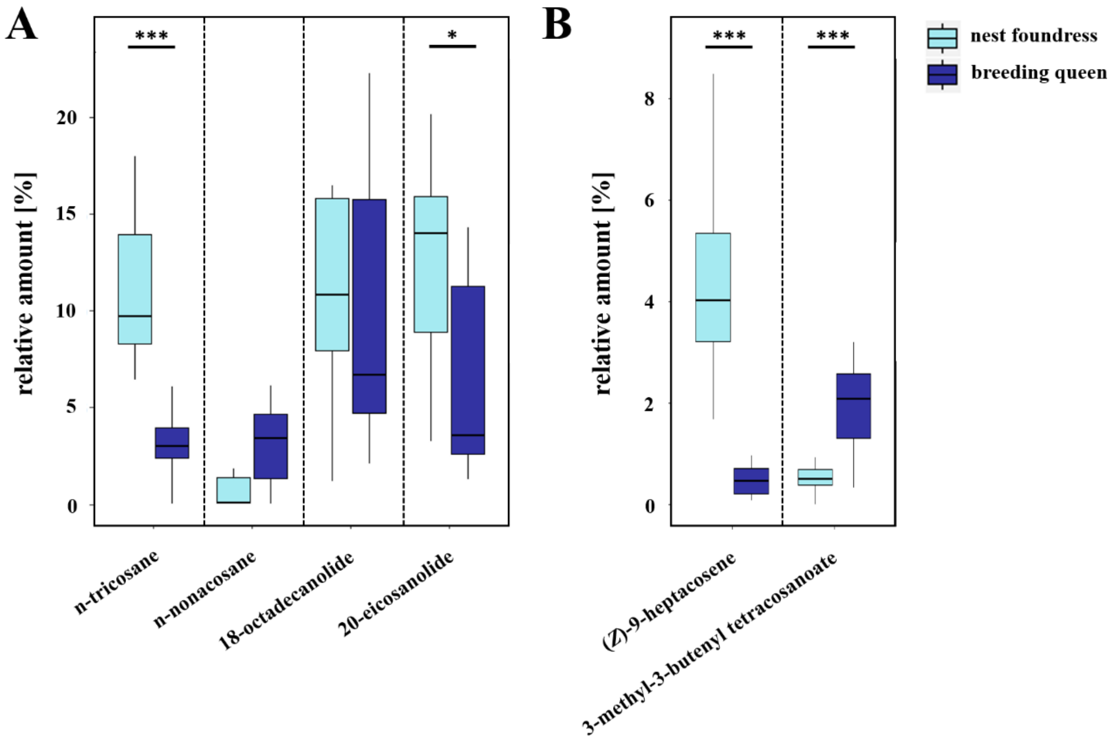

Figure S1: Comparison of relative amounts of (a) n-alkanes and macrocyclic lactones and (b) n-alkenes and isopentenyl esters on the cuticular surface which contributed more than 2.0 % to the Bray-Curtis dissimilarities between nest foundresses (cyan blue bars) and breeding queens (darkblue bars) of *L. pauxillum*. Bars represent the median as well as the 25th and 75th percentiles (asterisks indicate significant differences, Mann-Whitney-U tests: n-tricosane $P < 0.001$, 20-eicosanolide $P = 0.012$, (*Z*)-9-heptacosene $P < 0.001$, 3-methyl-3-butenyl tetracosanoate $P < 0.001$).

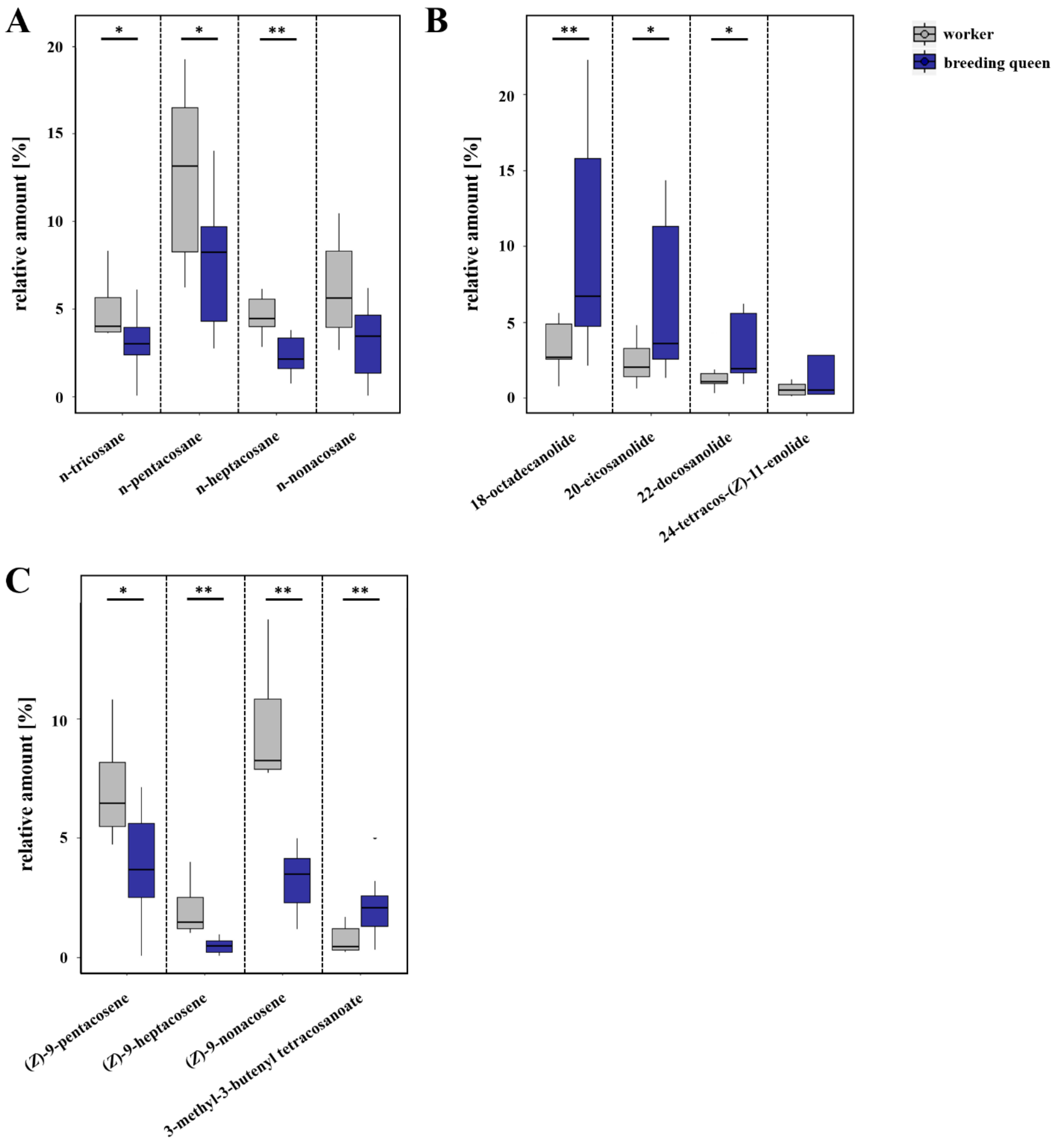

Figure S2: Comparison of relative amounts of (a) n-alkanes, (b) macrocyclic lactones and (c) n-alkenes and isopentenyl esters on the cuticular surface which contributed more than 2.0 % to the Bray-Curtis dissimilarities between workers (grey bars) and breeding queens (darkblue bars) of *L. pauxillum*. Bars represent the median as well as the 25th and 75th percentiles (asterisks indicate significant differences, Mann-Whitney-U tests: n-tricosane P = 0.026, n-pentacosane P = 0.033, n-heptacosane P = 0.001, 18-octadecanolide P = 0.009, 20-eicosanolide P = 0.047, 22-docosanolide P = 0.021, (*Z*)-9-pentacosene , (*Z*)-9-heptacosene P = 0.002, (*Z*)-9-nonacosene P = 0.005, 3-methyl-3-butenyl tetracosanoate P = 0.007).

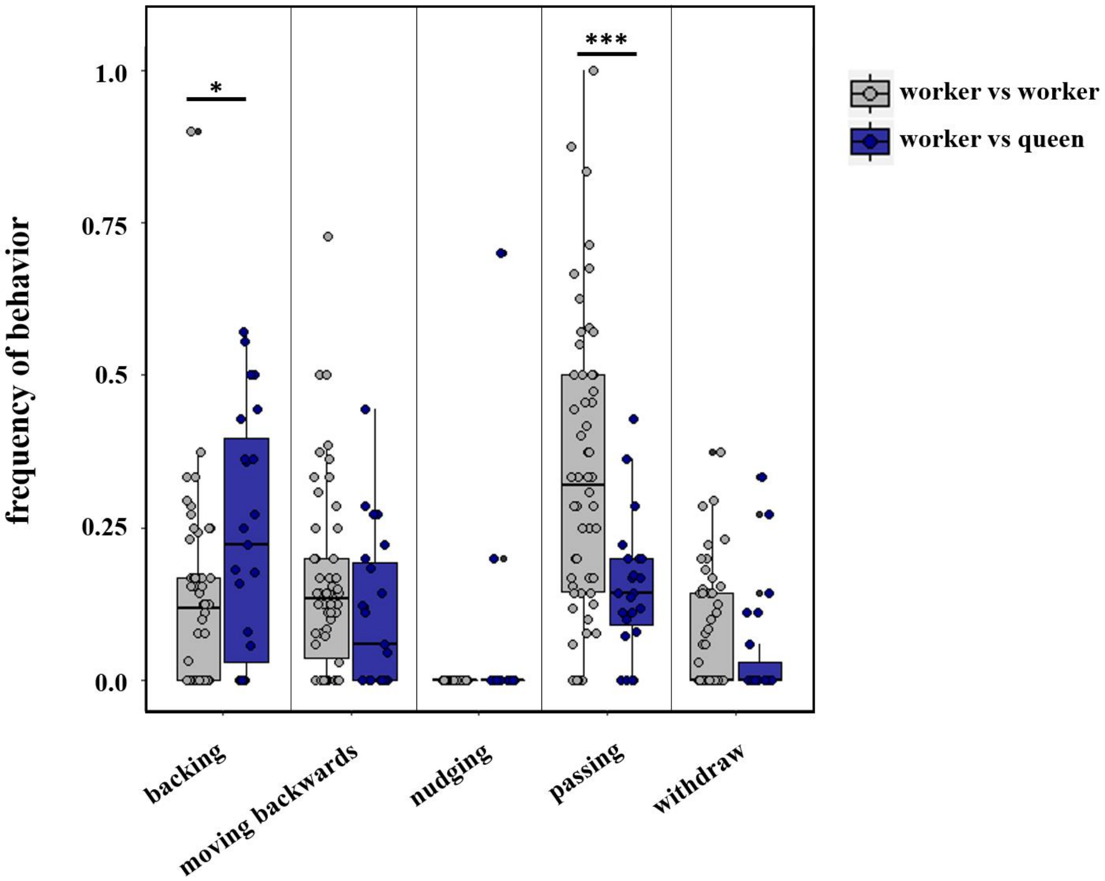

Figure S3: Comparison of worker behavior when interacting with another worker (grey bars) or with a queen (blue bars). Bars represent the median as well as the 25th and 75th percentiles. Workers showed a higher frequency of passing behavior (Mann-Whitney-U test, $P < 0.001$) and backing behavior when interacting with a queen (Mann-Whitney-U test, $P = 0.011$). There was no significant difference in the frequencies of the other observed behaviors (Mann-Whitney-U tests; $P > 0.05$). These results indicated that a high frequency of backing behavior is an indicator for queen recognition in *L. pauxillum* workers.

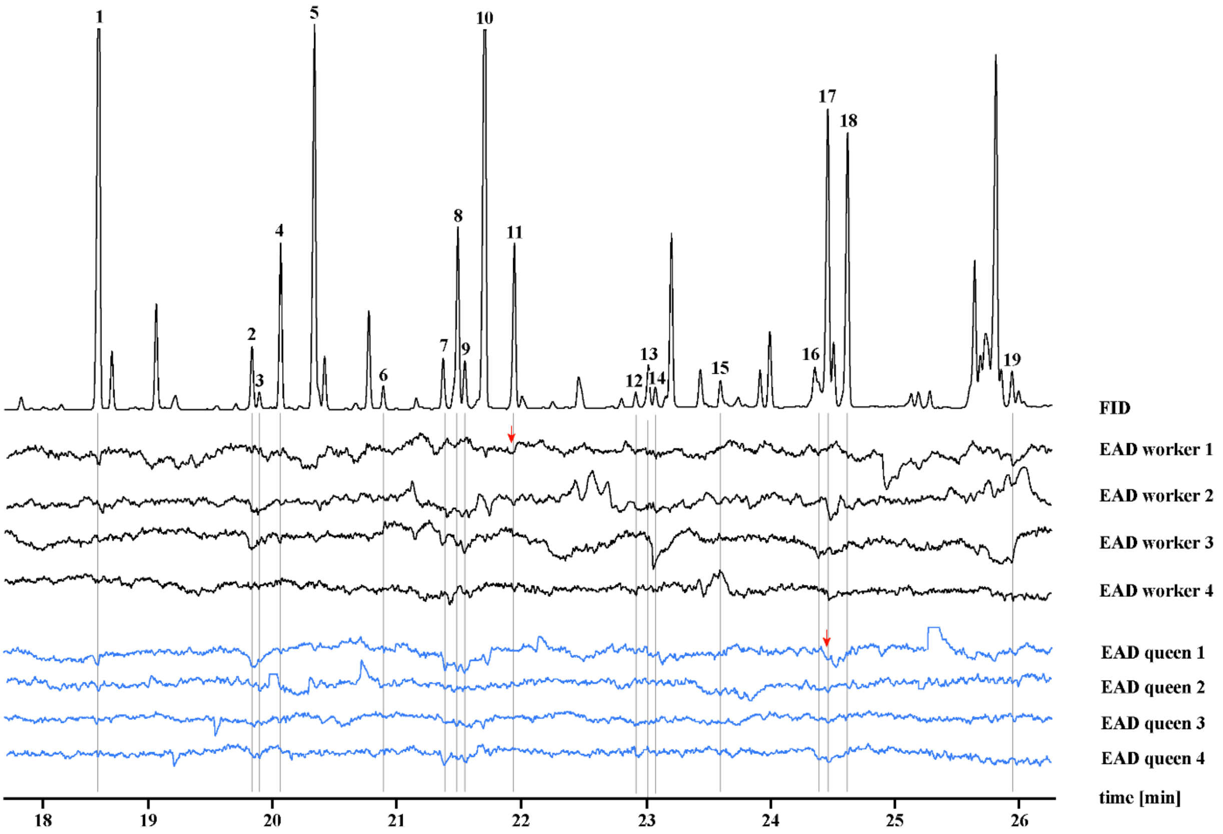

1) 18-Octadecanolide
2) (*Z*)-9-Tricosene
3) (*Z*)-7-Tricosene
4) Tricosane
5) 20-Eicosanolide
6) Tetracosane
7) 3-Methyl-3-butenyl octadecanoate
8) (*Z*)-9-Pentacosene
9) (*Z*)-7-Pentacosene
10) Pentacosane
11) 22-Docosanolide
12) (*Z*)-9-Heptacosene
13) (*Z*)-7-Heptacosene
14) 3-Methyl-2-butenyl eicosanoate
15) Octacosane
16) (*Z*)-9-Nonacosene
17) (*Z*)-7-Nonacosene
18) Nonacosane
19) Hentriacontane

.

Figure S4: Examples of coupled gas chromatographic and electroantennographic detection (GC-EAD) in cuticle surface extracts of *L. pauxillum* queens by using four different antennae of *L. pauxillum* workers (black) and queens (blue). Numbers and grey lines indicate compounds that were electrophysiologically active (EAD-active) in at least 4 runs of workers or queens. Red arrows indicate those compounds that were perceived only by either workers or queens.

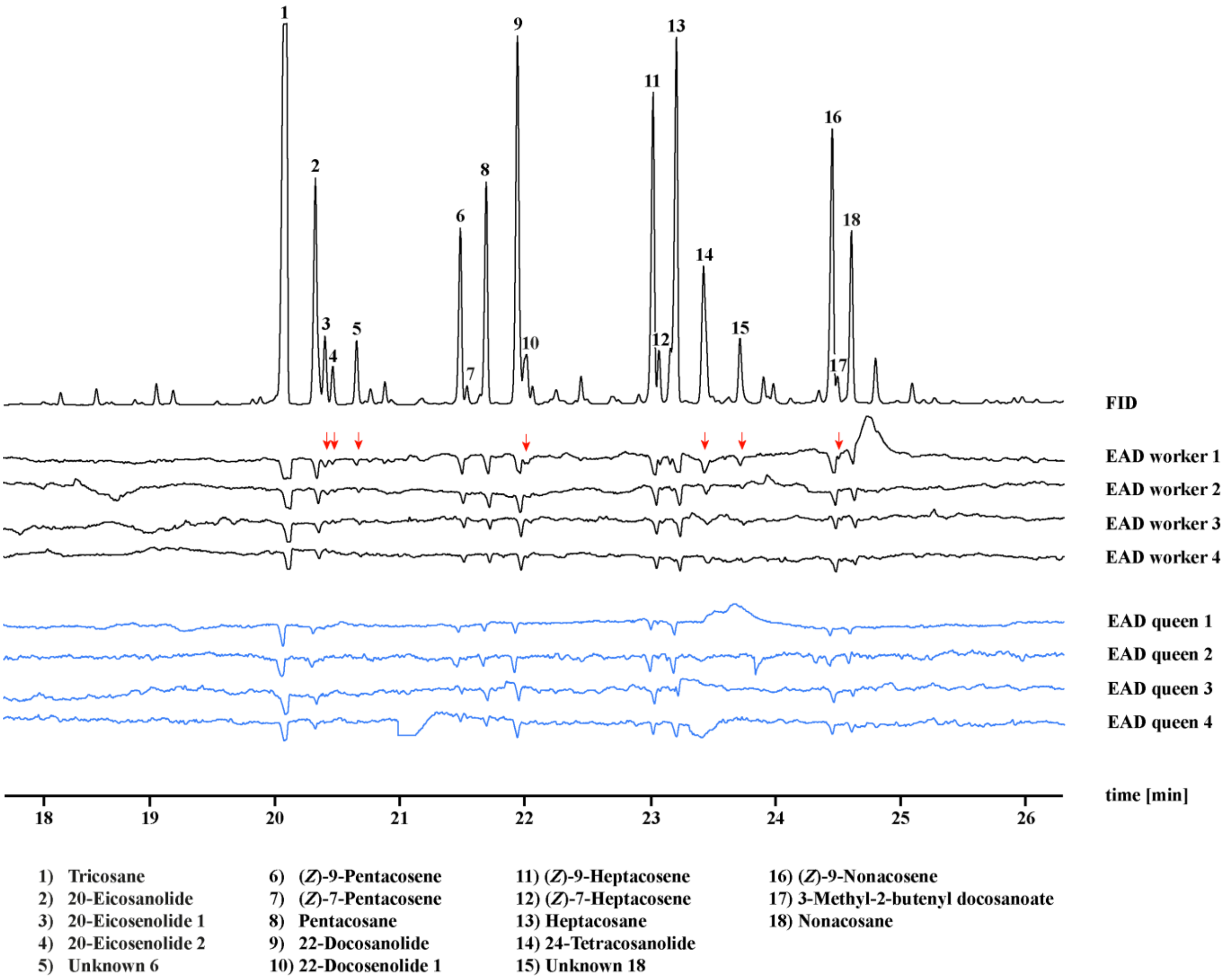

Figure S5: Examples of coupled gas chromatographic and electroantennographic detection (GC-EAD) in cuticle surface extracts of *L. malachurum* queens by using four different antennae of *L. malachurum* workers (black) and queens (blue). Numbers indicate compounds that were electrophysiologically active (EAD-active) in at least 4 runs of workers or queens. Red arrows indicate those compounds that were perceived only by workers.
